# 35058 A Mixed Method Study: Can Lyft Facilitate Better Access to Healthy Food?

**DOI:** 10.1017/cts.2021.592

**Published:** 2021-03-30

**Authors:** Raneitra Grover, Lenwood Hayman

**Affiliations:** Morgan State University School of Community Health and Policy

## Abstract

ABSTRACT IMPACT: Interventions designed to improve access to healthy food are needed as a mechanism to improve diet quality and ultimately prevent diet-related chronic diseases. OBJECTIVES/GOALS: For people living in food deserts, access to grocery stores is an important consideration toward improving diet quality. The Grocery Access Program (GAP) provided discounted Lyft rides to grocery stores for residents in Baltimore, MD. This study will assess how the GAP impacted access to different food retail stores and healthy food purchases. METHODS/STUDY POPULATION: A mixed methods sequential explanatory design will be used. We collected survey data at baseline and mid-pilot on primary grocery shopping store, frequency of purchasing fruits and vegetables, and frequency of using the discounted Lyft rides for 90 program enrollees. The Healthy Food Availability Index (HFAI), a validated, observation-based instrument, will be used to measure healthy food availability at participants’ primary grocery shopping stores; HFAI scores range from 0-27. We will also compare frequency of self-reported fruit and vegetable purchases before and after GAP participation. Quantitative data analysis using paired sample t-tests and chi square tests will be followed by in-depth interviews with GAP participants; thematic analysis will be used to analyze qualitative data. RESULTS/ANTICIPATED RESULTS: This research is in progress; survey data collection is complete and store-level HFAI data collection will begin soon. We hypothesize that GAP participants will have shopped at stores with higher HFAI scores than non-participants, and that participants will have purchased healthy foods more frequently than they did prior to GAP participation. If these hypotheses are not supported, our qualitative findings will elucidate potential reasons and mechanisms for improving the program. If our hypotheses are supported, it will provide evidence for the GAP as a convenient, low-cost intervention to improve healthy food access for people in low-income communities. DISCUSSION/SIGNIFICANCE OF FINDINGS: Access to healthy foods is an important social determinant of health, and innovative strategies that can facilitate better dietary habits are needed in the area of food access research. Findings from this study could be used to scale up efforts that will foster better food access, healthier diets, and ultimately better health outcomes.

